# Evaluation of Suspected Aspirated Beverage Can Pull Tab: Radiographs May Not Be Enough

**DOI:** 10.1155/2014/196960

**Published:** 2014-12-14

**Authors:** Amy L. Kotsenas, Norbert G. Campeau, Richard A. Oeckler, Ronald S. Kuzo

**Affiliations:** ^1^Department of Radiology, Mayo Clinic, 200 First Street SW, Rochester, MN 55905, USA; ^2^Division of Pulmonary & Critical Care Medicine, Department of Medicine, Mayo Clinic, Rochester, MN 55905, USA; ^3^Department of Physiology & Biomedical Engineering, Mayo Clinic, Rochester, MN 55905, USA

## Abstract

A 67-year-old male presented to the emergency department with concern for accidental aspiration of an aluminum beverage can pull tab. Neck and chest radiographs did not reveal an aspirated foreign body. Despite ongoing complaint of dysgeusia and adamancy of aspiration by the patient, he was discharged to home without recommendation for further follow-up. Seven months later, a computed tomography (CT) scan of the chest performed as part of an unrelated lung cancer work up confirmed the presence of a left mainstem bronchus metallic foreign body compatible with a pull tab. This case report illustrates the poor negative predictive value of radiographs for a suspected aluminum foreign body and demonstrates the superiority of CT for this purpose. In such presentations it is imperative to have a low threshold for performing further diagnostic evaluation with CT due to the relatively high radiolucency of aluminum.

## 1. Introduction

Conventional radiography is commonly used to evaluate the presence and location of metallic foreign bodies due to the erroneous assumption that all metal is readily demonstrated by this technique [1–5]. While true for metals used in coins or projectiles such as bullets, some—including aluminum—have very low X-ray attenuation and are often inconspicuous on conventional radiographs. Medical professionals are frequently unaware of the relatively high radiolucency of aluminum. Here we report a case of delayed diagnosis of an aspirated aluminum beverage pull tab in the central airways of an adult patient due to overreliance on radiographs.

## 2. Case Report

**Figure 1:**
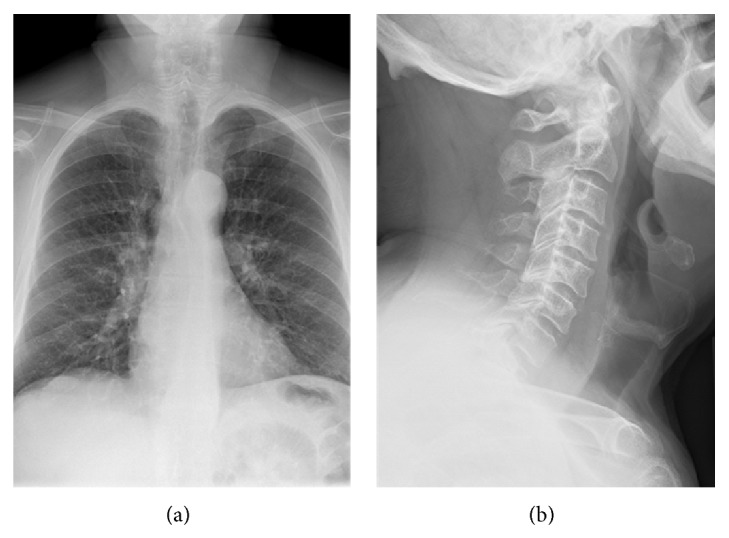
PA chest (a) and lateral neck (b) radiographs obtained day of suspected aluminum tab aspiration fail to demonstrate a metallic foreign body. There is no evidence for supportive findings on chest radiographs such as atelectasis or air-trapping.

A 67-year-old male former smoker sought medical attention following an episode in which he believed he accidentally aspirated an aluminum pull tab from a beverage can. Chest and neck radiographs (Figures 1(a) and 1(b)) did not reveal evidence of an aspirated foreign body, and the patient was told by his physician that he had likely ingested and “passed it.” The patient denied excreting the tab and maintained that he had a persistent metallic taste in his mouth that he believed was due to the presence of the aluminum beverage pull tab. The treating physician believed that a negative chest radiograph was sufficient to exclude the possibility of an aspirated aluminum pull tab, and therefore no further evaluation was performed and the patient was sent home without further follow-up.

**Figure 2:**
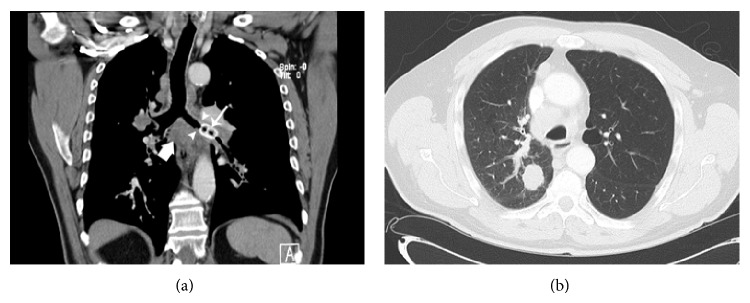
Reformatted coronal CT MIP image in soft tissue window-level setting (a) demonstrates presence of a hyperdense foreign body (solid arrow) within a thickened left mainstem bronchus (arrowheads). Also noted is subcarinal (block arrow) and hilar adenopathy which was later shown to represent nodal metastasis from lung adenocarcinoma. Axial CT imaging in lung window-level setting (b) shows resultant hyperinflation and hyperlucency of the left upper lobe related to air-trapping.

Approximately seven months later the patient developed increasing shortness of breath requiring hospitalization. A CT scan of the chest performed during this time demonstrated a large right upper lobe lung mass with associated hilar and mediastinal lymphadenopathy. Also confirmed was the presence of a metallic foreign body in the left mainstem bronchus (Figure 2(a)). The foreign body was associated with inflammatory changes, bronchial wall thickening, and severe narrowing of bronchial lumen. The left lung was hyperinflated and hyperlucent consistent with central airway obstruction and air-trapping (Figure 2(b)). The thin pull tab probably did not cause complete obstruction of the bronchus at the time of initial evaluation; however, the presence of the foreign body in the airway for several months likely led to inflammation and thickening of the surrounding bronchial wall and the development of severe stenosis or obstruction of the airway lumen. The patient underwent bronchoscopy and transbronchial biopsies of the enlarged subcarinal lymph nodes demonstrated adenocarcinoma. The aluminum beverage pull tab was extracted from his left mainstem bronchus during bronchoscopy.

## 3. Discussion

Aspiration of a foreign body is a common problem and can be a life-threatening emergency requiring bronchoscopic removal [6]. Because of a common misconception that all metal is radiopaque on radiographs, the conclusion of a negative search for an aluminum foreign body may be erroneously reached based upon conventional radiographs alone resulting in serious sequelae [7]. Premature discontinuation of a foreign body workup is less likely to happen with a history of radiolucent foreign body such as an inhaled peanut or piece of plastic, as these objects are not expected to be radiopaque, and subsequent evaluation with CT or bronchoscopy is readily performed.

Aluminum has low radiodensity and consequently may be inapparent on radiographs. The principle physical process responsible for the absorption of X-rays in soft tissue is the photoelectric effect, with X-ray absorption varying as *Z* ^3^/*E* ^3^, where *Z* is the atomic number of the object and *E* is the energy of the X-ray beam [8, 9]. For aluminum *Z* = 13, in comparison to *Z* = 26 for iron and *Z* = 82 for lead. The atomic number for aluminum is intermediate between bone (calcium, *Z* = 20) and soft tissue *Z* = 7.5 [4].

Additional physical properties such as thickness, density, and geometric position can also influence the radioopacity of a foreign object. Consequently, radiographic identification of small or thin pieces of swallowed or aspirated aluminum foreign bodies may be challenging. Foreign bodies imaged en face may be more difficult to detect than those oriented along the line of the beam. Finally, the location of the foreign body may contribute to its nonvisualization on radiographs: superimposition over osseous structures, such as the spine, can obscure detection [5].

CT is vastly superior to radiographs for detection of radiolucent foreign bodies and should be considered the “gold” standard [10, 11]. Even in retrospect, the aluminum pull tab could not be convincingly identified on either AP or lateral projections of the chest radiograph. Furthermore, CT is useful in demonstrating the precise location of the foreign body prior to bronchoscopy [9]. The utility of conventional radiography for detection of aluminum foreign bodies placed in upper esophageal and posterior pharyngeal area was evaluated in a controlled manner in ten randomly selected cadavers [5]. This series showed that the high positive predictive value of positive radiographic findings was sufficient to direct therapy without further evaluation. However, negative radiographs were deemed inadequate to completely rule out the presence of an aluminum foreign body and for such cases, further evaluation is necessary.

In conclusion, radiographs can be helpful if they identify the foreign body; however, caution is needed, as negative radiographs do not necessarily exclude presence of a foreign body. For cases of suspected aluminum foreign body aspiration, it is essential to have a low threshold for performing further evaluation with CT due to the relatively high radiolucency of aluminum.

## References

[1] Hunter T. B., Taljanovic M. S. (2003). Foreign bodies. *Radiographics*.

[2] Lan R.-S. (1994). Non-asphyxiating tracheobronchial foreign bodies in adults. *The European Respiratory Journal*.

[3] Ross M. N., Janik J. S. (1988). ‘Foil tab’ aspiration and retropharyngeal abscess in a toddler. *Journal of the American Medical Association*.

[4] Stewart G. D., Lakshmi M. V., Jackson A. (1994). Aluminium ring pulls: an invisible foreign body. *Journal of Accident and Emergency Medicine*.

[5] Valente J. H., Lemke T., Ridlen M., Ritter D., Clyne B., Reinert S. E. (2005). Aluminum foreign bodies: do they show up on X-ray?. *Emergency Radiology*.

[6] Baharloo F., Veyckemans F., Francis C., Biettlot M.-P., Rodenstein D. O. (1999). Tracheobronchial foreign bodies: presentation and management in children and adults. *Chest*.

[7] Al-Majed S. A., Ashour M., Al-Mobeireek A. F., Al-Hajjaj M. S., Alzeer A. H., Al-Kattan K. (1997). Overlooked inhaled foreign bodies: late sequelae and the likelihood of recovery. *Respiratory Medicine*.

[8] Bushberg J. T. (2002). *The Essential Physics of Medical Imaging*.

[9] Koşucu P., Ahmetoǧlu A., Koramaz I., Orhan F., Özdemir O., Dinç H., Ökten A., Gümele H. R. (2004). Low-dose MDCT and virtual bronchoscopy in pediatric patients with foreign body aspiration. *The American Journal of Roentgenology*.

[10] Berger P. E., Kuhn J. P., Kuhns L. R. (1980). Computed tomography and the occult tracheobronchial foreign body. *Radiology*.

[11] Kjhns L. R., Borlaza G. S., Seigel R. S., Paramagul C., Berger P. E. (1979). An in vitro comparison of computed tomography, xeroradiography, and radiography in the detection of soft-tissue foreign bodies. *Radiology*.

